# Antimicrobial peptide AMP-17 induces protection against systemic candidiasis and interacts synergistically with fluconazole against *Candida albicans* biofilm

**DOI:** 10.3389/fmicb.2024.1480808

**Published:** 2024-11-01

**Authors:** Chaoqin Sun, Lijuan Zhu, Longbing Yang, Zhuqing Tian, Zhenlong Jiao, Mingjiao Huang, Jian Peng, Guo Guo

**Affiliations:** ^1^School of Basic Medical Sciences, Key Laboratory of Microbio and Infectious Disease Prevention & Control, Guizhou Medical University, Guiyang, China; ^2^Key Laboratory of Environmental Pollution Monitoring and Disease Control, Ministry of Education, Guizhou Medical University, Guiyang, China; ^3^Center of Laboratory Medicine, The Affiliated Hospital of Guizhou Medical University, Guiyang, China; ^4^Department of Laboratory Medicine, Guizhou Provincial People’s Hospital, Guiyang, Guizhou, China; ^5^Translational Medicine Research Center, Guizhou Medical University, Guiyang, China

**Keywords:** *Candida albicans*, antimicrobial peptide, AMP-17, systemic candidiasis, synergistic effect, biofilm

## Abstract

*Candida albicans*, a common commensal and opportunistic fungal pathogen in humans, can occasionally progress to disseminated candidiasis which is a serious condition with a high morbidity and fatality rate. The emergence of drug-resistant fungal strains compels us to look for an efficient treatment solution. Our earlier studies have demonstrated that the unique antimicrobial peptide AMP-17 from *Musca domestica* has a strong antifungal impact on *C. albicans in vitro*. Here, we verified the therapeutic effects of AMP-17 on systemic candidiasis *in vivo* and the peptide interacts with fluconazole, a common antifungal medication, to treat systemic candidiasis. In the disseminated candidiasis model of *Galleria mellonella* and mice challenged with *C. albicans*, AMP-17 increased the survival rates of infected larvae and mice to 66.7 and 75%, respectively. Furthermore, the peptide lowered the load of *C. albicans* in the infected larvae and the kidneys of the mice by nearly 90%. Additional histological examination and measurements of plasma cytokines showed that the injection of AMP-17 markedly reduced the inflammatory response and balanced cytokine expression. Furthermore, checkerboard micro dilution experiments demonstrated that AMP-17 and fluconazole worked in synergy to inhibit *C. albicans* in the biofilm mode. According to morphological studies, AMP-17 and fluconazole together decreased the production of hyphae throughout the *C. albicans* biofilm formation process, loosening the mature biofilms’ structure and lowering the amount of carbohydrates in the extracellular matrix (ECM) of the biofilms. Taken together, these results showed that AMP-17 would be a viable treatment for systemic candidiasis and might be a different approach to combating *Candida* biofilm, either by itself or in conjunction with fluconazole.

## 1 Introduction

*Candida albicans*, a type of common nosocomial fugus, can attack almost any organ through hematogenous diffusion or overgrowth from colonized sites within the body, especially in immunocompromised patients. With a high occurrence of hematogenously disseminated candidiasis, *Candida* species which is frequently isolated from bloodstream in hospitals (Wisplinghoff et al., 2004; Wisplinghoff et al., 2014), has alarmingly high crude and attributable mortality rates ranging from 40 to 60%, even with antifungal drug therapy (Gudlaugsson et al., 2003; Morgan, 2005). According to the previous reports, the incidence of systemic candidiasis among invasive fungal infections in ICUs stands in first place (Bassetti et al., 2017). Furthermore, *Candida* species are mostly harmful due to their ease of adhesion to abiotic surfaces, ability to form filaments, and production of strong biofilms on medical equipment (Pereira et al., 2021). A biofilm is constituted by a community of microbes attached to a surface and packed in an extracellular matrix (ECM) (Nobile and Johnson, 2015; Pereira et al., 2021). It is the high density of cells and thick ECM in biofilms that protect the cells from environmental assaults, innate immune cells, and antifungal drug attacks (Sutar et al., 2022). So, the capacity of *C. albicans* to form biofilms exacerbates the infection and complicates therapy.

Nowadays, three classes of agents (azole, echinocandin, and polyene) are often applied to treat invasive candidiasis. Nevertheless, the widespread use of fluconazole, the first-line antifungal drug in clinics, has trigged the emergence of fluconazole-resistant *C. albicans*, sparking a worldwide medical attention. Currently, the optional therapy for fungal infections is confined and offers little diversity in their mode of action. Additionally, the similarities between fungal and mammalian cells at the biochemical and biological levels challenged the tissue (Cowen, 2008). This, coupled with the absence of new antifungal drugs entering the market over the past decade, has necessitated the urgent introduction of novel antifungal agents. Recently, antimicrobial peptides (AMPs) have occupied much consideration as new candidates to traditional antibiotics (Rezende et al., 2021). AMPs derived from natural defense system of hosts represent a crucial element of the innate immune system, serving as the primary defense against pathogens (Diamond et al., 2009). A common feature of AMPs across all phyla is that they are typically cationic and amphipathic (Rezende et al., 2021). These two features empower the peptides with a wide array of activities, as they facilitate their interactions with the entire cellular membrane or through other intricate mechanisms (Chan et al., 2006). Consequently, there is a decreased chance of developing antimicrobial resistance since the pathogen’s membrane charge is less likely to change (Chan et al., 2006). Other than their antimicrobial characteristics, AMPs can also take part in immunomodulatory and wound healing (Yang et al., 2020).

We have previously investigated a novel antimicrobial peptide AMPs-17 from *Musca domestica* with multi-antifungal effects such as *Candida*. spp., *Cryptococcus*. spp., and *Aspergillus* spp. (Guo et al., 2017; Ma H. et al., 2020; Zhu et al., 2022). For the peptide acquired after *Musca domestica* challenged with *C. albicans*, further studies demonstrated AMP-17 indeed exerted antifungal activity against *C. albicans* specifically through disrupting cell membrane integrity and disturbing intracellular oxidative reactions (Ma H. et al., 2020; Ma et al., 2023; Yang et al., 2023). In addition, the yeast cells became small, and hyphal elongation stopped when exposed to AMP-17, finally leading to defective biofilms (Guo et al., 2017; Sun et al., 2022). Importantly, the anti-biofilm capacity of AMP-17 could potentially provide a promising alternative for *Candida*. spp. biofilm-related drug resistance in clinical settings. Reportedly, the AMPs, with a wide spectrum of antimicrobial and anti-biofilm activity, showed effective synergy against fluconazole-resistant strains when combined with fluconazole (Czechowicz et al., 2021). Previous research has shown that AMP-17 significantly inhibited both planktonic and biofilm *C. albicans*. This implies that AMP-17 could function as an enhancer or substitute for antifungal drugs in the treatment of fungal infections. Therefore, an increase in fluconazole sensitivity against *C. albicans* biofilms is anticipated in response to AMP-17. More significantly, it was still unknown if AMP-17’s *in vitro* antifungal potential could be further expanded to have a therapeutic impact against *Candida* infection *in vivo*.

In this work, we used the *Galleria mellonella* and mice model infected by *Candida albicans* to assess the therapeutic impact of AMP-17 against invasive candidiasis. Histopathological examination verified that AMP-17 prevented inflammatory damage in *C. albicans* infected mice by lowering plasma tumor necrosis factor (TNF)-*α* levels and the blood neutrophil ratio. Furthermore, when combined with fluconazole, AMP-17 demonstrated a great synergy against *C. albicans* biofilms. According to morphological tests, the combination of the peptide and fluconazole prevented the growth of hyphae, loosened the robust structure of the biofilm, and reduced the amount of carbohydrates in the extracellular matrix (ECM) of the *C. albicans* biofilm.

## 2 Materials and methods

### 2.1 *Candida* strains and culture conditions

*Candida albicans* reference strain SC 5314 and *Candida parapsilosis* ATCC 22019 were used in this work. And another 10 clinical isolates of *Candida albicans* used in this study were presented by the Dermatology Department of Peking University People’s Hospital, including 3 strains (16105, 16102, 16138) obtained from bronchial perfusate, 3 strains (16225, 16229, 16162) from sputum, 2 strains (16214, 16111) from faces, 1 strain (16228) from oral mucosa, and 1 strain (16230) from hydrothorax. The strains were preserved in brain-heart infusion broth containing 20% glycerol at −80°C in the Key Laboratory of Modern Pathogenic Biology of Guizhou Medical University. Before experiments, the frozen strains were revived on SDA plates (4% dextrose, 1.8% agar, and 1% peptone), and subsequently nurtured in YPD broth (2% glucose, 2% peptone, and 1% yeast extract) at 30°C for 12 h with shaking at 200 rpm to grow to the logarithmic phase. While, for biofilm formation, a final concentration of 10% fetal bovine serum (Sigma-Aldrich, St. Louis, USA) was added to RPMI-1640 culture (Invitrogen, Carlsbad, USA).

### 2.2 AMP-17 and antifungal drugs

The antimicrobial peptide AMP-17 was produced using a prokaryotic expression system according to previously described methods (Sun et al., 2022; Yang L. B. et al., 2022). Briefly, the cloned gene of full-length AMP-17 was inserted into a His-tagged *Escherichia coli* pET-28(+) to express the recombinant peptide. Then, the recombinant peptide AMP-17 underwent a Ni-NAT HisTrap FF column of chromatography (Novagen, Germany) for purification, followed by the elimination of imidazole by an ultrafiltration tube (Millipore, Amicon^®^Ultra, USA). AMP-17 was granted a Chinese patent license in 2019 (patent number: ZL 2016 10428119.8), with the amino acid sequence detailed in our previous study (Yang L. B. et al., 2022). The typical antifungal agent fluconazole (FLC) (Sigma, USA) in clinical therapy was used as a positive drug for comparing the antifungal activity, and also as the combined objective to evaluate the synergistic efficacy with AMP-17. A stock solution of antifungal drugs was prepared in 1% dimethyl sulfate (DMSO) at a concentration of 2560 μg/mL and store at −20°C.

### 2.3 *Galleria mellonella* model of systemic candidiasis

To evaluate the antifungal effect of AMP-17 *in vivo*, we employed an established wax moth model system (Piatek et al., 2020). *Galleria mellonella* larvae (Huiyude Biological Technology, Tianjin, China) with 250 ~ 300 mg of body weight were distributed as different groups with randomly selected larva (*n* = 20/group). The *C. albicans* SC 5314 were grown to exponential phase after overnight culture, then washed three times with sterile PBS, finally suspended in PBS at the concentration of 5 × 10^7^ CFU/mL. Experimental groups comprised as follows: blank control group (PBS as vehicle), model group (fungal infection), and treatment groups. FLC was applied as a positive control. Each larva was injected with 10 μL of the prepared fungi on the last left proleg, and 1 h later AMP-17 at the concentration among 0.25–1 mg/kg was administered to the infected larvae, with all injections performed by a Hamilton syringe. The larvae were kept at 37°C for 5 days, with live and dead counts monitored every 24 h. Moreover, fungal burden and histopathological assessment of *G. mellonella* larvae were detected at 24 h post-treatment. For fungi burden, 6 larvae per group were randomly selected to grind with a high-speed homogenizer. Then, the homogenate was spotted on sterile solid YPD medium after serial 10 times dilution, and the fungal cells were not counted until the colonies presented on the culture medium. For histopathological assessment, the *G. mellonella* larvae were fixed with 4% paraformaldehyde, embedded in tissue-tek OCT, processed in cryosectioned tissue (10 μm), and then dehydrated in graded alcohol solutions, finally stained with periodic acid-Schiff (PAS) solution.

### 2.4 Murine model of disseminated candidiasis

To further investigate the antifungal efficacy of AMP-17 in a murine model, survival rate and histopathological assessments of the kidneys were examined in the BALB/c mice model of disseminated candidiasis (Wang et al., 2022). A total of 168 female mice aged 8–12 weeks were acquired from the Laboratory Animal Center of Guizhou Medical University and divided into four groups to establish the disseminated candidiasis model. All animal protocols were implemented in line with the NIH Guide for the Care and Use of Laboratory Animals and authorized by the Institutional Animal Care and Use Committee of Guizhou Medical University (No. 2000455). The BALB/c mouse model was induced via the lateral tail vein with 100 μL *C. albicans* suspension (2 × 10^6^ cells/mL) in all groups except the control group. Next, 10 mg/kg of AMP-17 and FLC were administered to the corresponding mice by intraperitoneal injection after inoculation for 24 h, while the control and model groups were injected with equal volumes of sterile saline. Treatments were continued for 14 days, and live and dead mice were monitored every day. The kidneys of 6 mice at 2^nd^ and 8^th^ day post-infection in each group were excised, sonicated to homogenate tissue. Then the kidney tissue suspension was serially diluted, pointed on SDA plates, cultivated overnight at 30°C and the colony-forming units (CFU) were counted. The fungal burdens of kidneys were calculated as CFU/g of the organ. Meanwhile, half of kidney samples underwent histopathological analysis, which was subjected to 10% neutral formalin, gradually dehydrated in alcohol solutions, embedded in paraffin, and stained with hematoxylin and eosin (H&E) or PAS solutions (Chen et al., 2020). At the end of the experiment, the plasma was separated from the blood by centrifugation at 1500 *g* for 10 min and preserved at −80°C. Subsequently, cytokine content was measured by the Mouse ELISA Kit (Gene Mei, Wuhan, China) following the manufacturer’s protocol.

### 2.5 Checkerboard assay

To explore the interaction of AMP and FLC, minimum inhibitory concentrations (MICs) of AMP-17 and AMP-17 plus FLC against *C. albicans* were assessed by using a broth microdilution method under the guidance of Clinical and Laboratory Standards Institute (CLSI) M27-S4 (CLSI, 2017). Briefly, 100 μL of a 2-fold dilution of AMP-17 was first added to the 96-well plate to achieve 2 to 512 μg/mL as an experimental concentration, and the final concentration of FLC ranged from 0.5 to 128 μg/mL. In addition, 100 μL of YPD medium was added as AMP-17 or FLC was performed alone. Next, 100 μL of yeast cell suspension of 1–5 × 10^3^ cells/mL was added to each well. Followed by incubation of the flat bottom 96-well plates at 35°C for 24 h to visually determine the MIC value defined as the lowest concentration that inhibits fungal growth by ≥80% compared to the corresponding drug-free growth control. Subsequently, the interaction between AMP-17 and FLC was analyzed by the checkerboard microdilution method as follows: AMP-17 underwent a 2-fold dilution across columns 1 to 11, whereas FLC was subjected to a 2-fold dilution from row A to G in the 96-well plate, and MICs of all combinations of AMP-17 and FLC at serial concentrations were obtained. Finally, the fractional inhibitory concentration (FIC) index was calculated using the formula as shown: FICI = (MIC_AMP-17 + FLC_/MIC_AMP-17_) + (MIC_FLC + AMP-17_/MIC_FLC_), where MIC_AMP-17 + FLC_ and MIC_FLC + AMP-17_ are combinations of test drugs, and MIC_AMP-17_ and MIC_FLC_ are valued for the test drugs alone. Accordingly, an FICI of 0.5 or less was defined as synergism, 0.5 < FICI <1.0 as partial synergistic, an FICI of 1.0 as an additive effect, 1.0 < FICI ≤4.0 as no interaction or inference, and FICI >4.0 as antagonism (Khodavandi et al., 2010). When the fluconazole (FLC) minimum inhibitory concentration (MIC) for *C. parapsilosis* ATCC22019 varied from 0.5 to 4.0 micrograms per milliliter, the experimental data were considered reliable. Experiments were independently conducted three times.

### 2.6 Antibiofilm activity

The capacity of AMP-17 to inhibit *C. albicans* biofilm was evaluated by 2,3-bis-(2-methoxy-4-5-sulfophenyl)-2H-tetrazolium-5-carboxanilide (XTT) with menadione, which measures metabolic activity. 200 μL volumes of *C. albicans* suspension (10^6^ CUF/mL) were deposited onto a 96-well plate and incubated at 37°C for 2 h until fully cell adhesion. Next, the medium was carefully sucked away and treated with AMP-17, FLC, and AMP-17 plus FLC for 12 h. After treatment, the planktonic cells were removed from the wells of the plate, which was then washed three times with sterile PBS. Next, 150 μL of XTT (Yuanye Co., Ltd., Shanghai, China) solution was introduced into each well, followed by a 30-min incubation at 37°C in the dark and the biofilm was washed with PBS for three times. Subsequently, 100 μL of supernate were transferred to a new 96-well plate, and OD_490_ nm was measured by a microplate reader (Bio-Rad, USA). And the sessile minimum inhibitory concentration (SMIC) and the sessile minimum eradication concentration (SMEC) refer to the lowest concentration that inhibits biofilm formation or eradicates mature biofilm by at least 80% compared to the control without drugs. Furthermore, to evaluate the capacity of AMP-17 plus FLC to damage the mature biofilms, *C. albicans* was first cultured in RPMI-1640 medium for 24 h to form biofilms and then subjected to the above drugs for another 24 h. The viability of biofilm was assessed by XTT assay and morphological analysis by SEM and CLSM analysis. Each experiment was independently carried out in triplicate.

For the interaction of AMP-17 and FLC against *C. albicans* biofilms, a checkerboard microdilution assay was also employed to determine the value of SMICs and SMECs of tested drugs with various combined modes. Then the calculation method of FICI for the biofilm of *C. albicans* is the same as that for planktonic bacteria. As well, the same cutoff scope of FICI was adopted to judge the interaction of AMP-17 and FLC on biofilm formation and destruction. Each experiment was independently performed, at least in triplicate.

### 2.7 Observation of *Candida albicans* biofilm

The effect of AMP-17 plus FLC on structural changes of *C. albicans* biofilm was visualized by scanning electron microscopy (SEM) and confocal laser scanning microscopy (CLSM). Notably, one *C. albicans* strain on which AMP-17 demonstrated synergistic effect with FLC was randomly selected for the morphological analysis. Briefly, *C. albicans* biofilms were formed on glass coverslips in the 6-wall plate at 37°C in the RPMI-1640 medium as described previously and treated with combined drugs at the concentrations of SMIC or SMEC, as well as the single drug with the equal concentration. After treatment, the supernatant was removed, and the coverslip was washed with PBS for three times. Afterwards, the coverslip was fixed with 2.5% (v/v) glutaraldehyde solution for 4 h, and then gradually dehydrated in ethanol at concentrations of 25, 50, 75, 95, and 100%. Finally, the *C. albicans* biofilm was dried and sprayed with gold and captured by a SU8100 scanning electron microscope (HITACHI, Japan).

In another separate experiment, *C. albicans* were cultured in the 6-wall plate, and biofilms formed on the sterile flips with the same treatment as previously described. After treatment, the supernatant was sucked away, and the flips were washed with sterile PBS three times. Subsequently, the biofilm was stained with 10 mM SYTO 9 (Invitrogen, USA) and 10 mM PI (Sigma, St. Louis, MO, USA) at 37°C for 30 min in the dark. Next, the dry solution was removed, and the flips were washed three times to get rid of the unbound dry. Then the flips were attached to the polyresistant acid-treated slides, and the confocal photos were taken by a SpinSR10 Confocal Microscope (Olympus, Japan) at an excitation wavelength of 488 nm for SYTO9 and 525 nm for PI.

### 2.8 ECM extraction and carbohydrate analysis

For ECM extraction, *C. albicans* biofilms were individually cultivated in 6-well plates by incubating the cells in RPMI for 48 h. Subsequently, the control samples were supplemented with fresh RPMI media, while another set of samples underwent treatment for 24 h, including 32 μg/mL AMP-17, 32 μg/mL fluconazole, and a combination of AMP-17 and fluconazole with equal concentrations. The wells were washed three times to remove the media, and the biofilms were scraped from the well surfaces into sterile PBS using a scraper (Gupta et al., 2021b). The extracted biofilm was sonicated at 35 W on an ice bath for 5 cycles (30 s each), and then the suspension was centrifuged for 5 min at 12000 rpm, and the supernatant was collected for carbohydrate analysis. Untreated *C. albicans* biofilm was regarded as controls, and drug-treated samples as targets.

The carbohydrate in ECM was detected using the phenol and sulfuric acid method (Gupta et al., 2021a). The sample was mixed with 5% phenol and pure H_2_SO_4_. Followed by incubating the samples for 10 min at room temperature and a thermal treatment at 30°C for 30 min. Finally, the OD_490nm_ of samples was measured, and carbohydrate content in the ECM was determined as μg/mL using a linear standard curve of glucose.

### 2.9 Statistical analyses

Each experiment was performed with independent biological replicates. Statistical analyses were considered by one-way ANOVA, and survival rates were analyzed with the log-rank test using GraphPad Prism 9 software (GraphPad Software Inc., La Jolla, CA, USA). *p* value 0.05 was considered statistically significant.

## 3 Results

### 3.1 The therapeutic effect of AMP-17 in a *Galleria mellonella* model of systemic candidiasis

**Figure 1:**
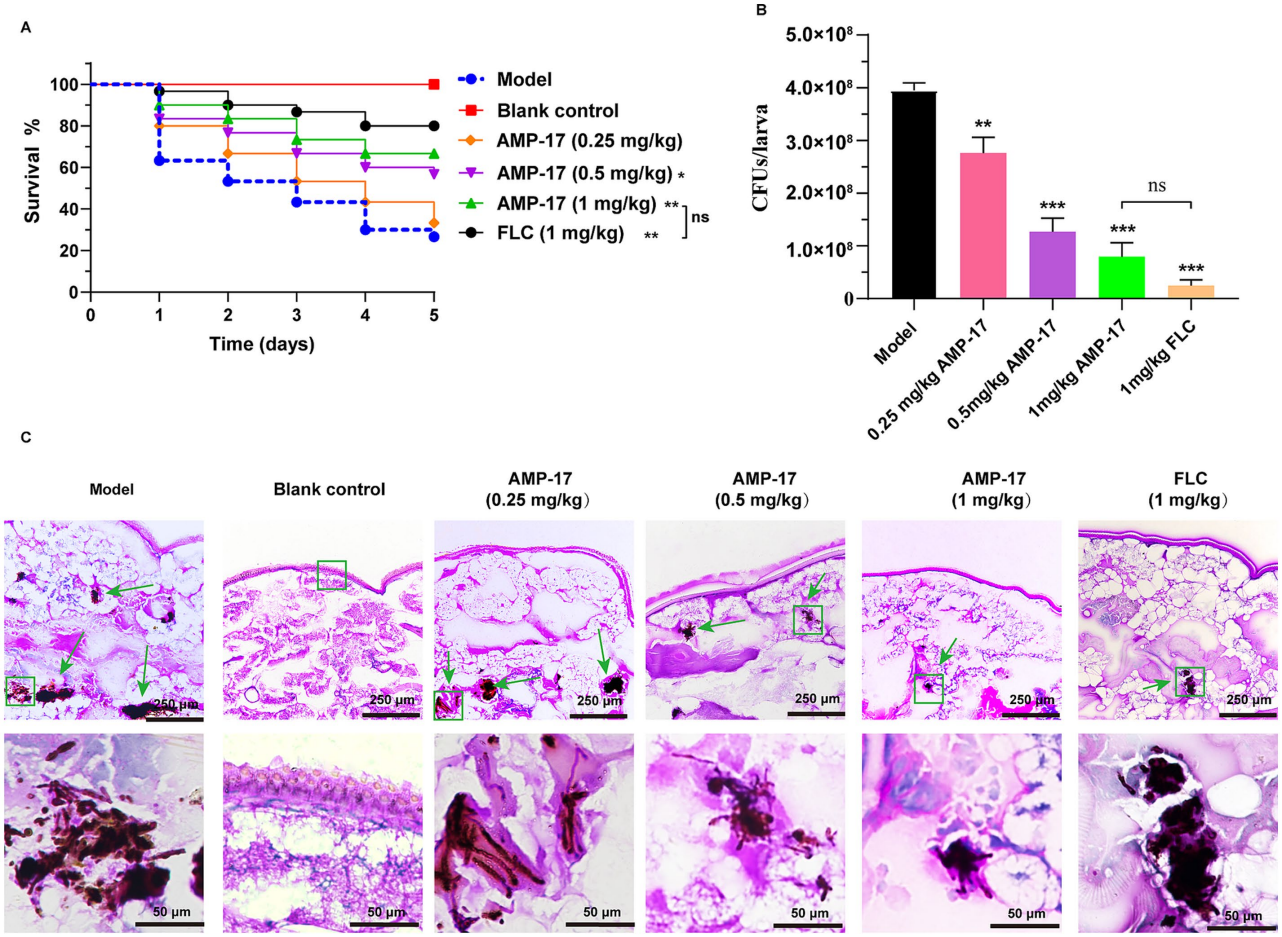
The therapeutic of AMP-17 on systematic candidiasis in *G. mellonella* model. The survival **(A)** and fungal loads **(B)** in the *G. mellonella* larvae challenged with a sublethal dose of 0.5 × 10^6^ *C. albicans*/larva and the therapeutic effect of AMP-17 with a range concentration of 0.25 to 1 mg/kg. FLC at 1 mg/kg as the positive agent in the parallel experiments. Larvae treated at 1 mg/kg showed 67.67% survival on the 5th day post-infection, with a reduction of more than 90% fungal load. Analysis was performed by analysis of variance (ANOVA): Ns: *p* > 0.05, *p < 0.05, **p < 0.01, ***p < 0.001, compared with the model group. **(C)** PAS staining of *G. mellonella* larvae after AMP-17 treatment. The green box and arrow indicated *C. albicans* filaments and yeast in the tissue of the larvae. Bar, 100 μm.

It is reported that *G. mellonella* has been demonstrated to be a suitable organism for assessing the impact of antifungal medications and novel substances with antifungal attributes on host-fungus interactions (Kavanagh and Sheehan, 2018). In the present study, the *G. mellonella* model was applied to evaluate the *in vivo* effects of AMP-17 on systemic candidiasis. As is shown in Figure 1, AMP-17 displayed a significant therapeutic effect on larvae infected with *C. albicans*. Survival curve showed that more than two-thirds of larvae died during 5 days after administration of *C. albicans* at the sublethal dose (1 × 10^7^ CFUs in each larva). However, after G. *mellonella* larvae were infected with *C. albicans*, we treated them with 3 different concentrations of AMP-17. Importantly, we observed that AMP-17 at concentrations of 0.5 and 1 mg/kg significantly improved the survival of *C. albicans* infected larva when compared to the untreated group. Specifically, the group treated with 1 mg/kg AMP-17 had 66.7% survival on the 5th day post-infection, exhibiting a similar therapeutic effect with the same concentration of FLC.

Next, the fungal burden of *G. mellonella* larvae after treatment for 5 days was calculated to verify the therapeutic efficacy of the peptide. Simultaneously, on the 5th day post-infection, treatment of AMP-17 at all the concentrations significantly decreased the fungal burden in the larva tissue. Furthermore, nearly 90% of *C. albicans* colonies in each larva reduced after 1 mg/kg AMP-17 treatment, which decreased fungal burden from 3.95 × 10^8^ CFUs/larva to 5.61 × 10^7^, showing no significance with 1 mg/kg FLC (Figure 1B). These findings were supported by a semi-quantitative analysis of larval tissue histological sections. AMP-17 at 0.5 and 1 mg/kg significantly reduced fungal load and dissemination in the larvae, along with a decrease in larval tissue melanization compared to the untreated group (Figure 1C). Simultaneously, only a limited number of melanization clumps exhibited visible rose-red fungal cells in the AMP-17-treated larvae. These data suggested that AMP-17 possessed a notable antifungal efficacy against *C. albicans in vivo*.

### 3.2 AMP-17 protected mice from disseminated candidiasis

**Figure 2:**
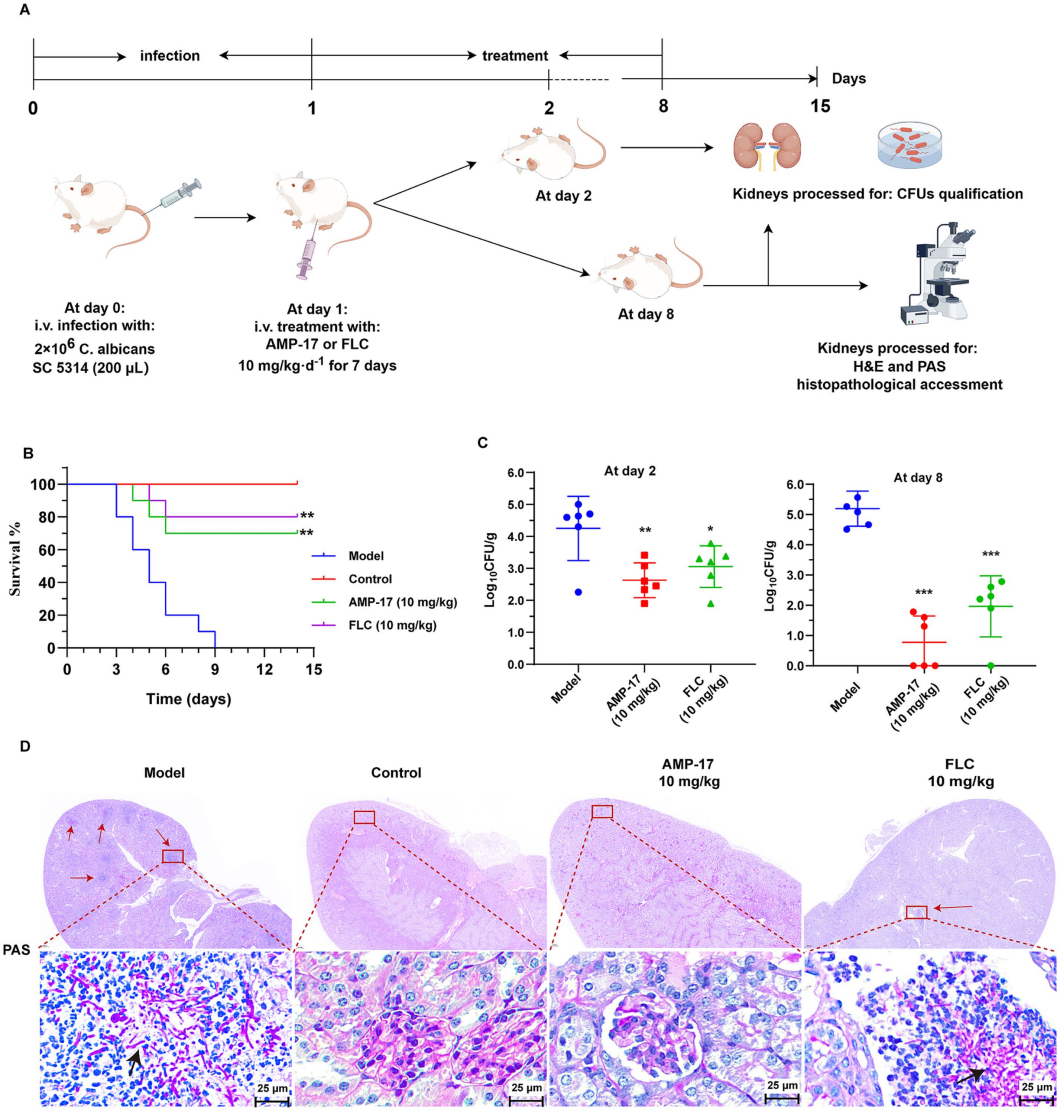
The *in vivo* antifungal efficacy of AMP-17 in the mice model of disseminated candidiasis. **(A)** Schematic process of treatment with AMP-17 and FLC in the murine model of disseminated candidiasis. AMP-17 and FLC at the dose of 10 mg/kg was intraperitoneally injected daily for the therapy of candidiasis mice induced by intravenous injection of 2 × 10^6^ *C. albicans*. **(B)** The Kaplan–Meier survival curves of mice monitored for 8 days post-inoculation. *N* = 10 mice per group. Survival rate of mice was carried out by the log-rank test. **(C)** Colony-forming units (CFUs) of *C. albicans* in kidneys from AMP-17 or FLC treated mice administered though intravenous injection with *C. albicans* post inoculation 2 and 8 days. *N* = 6 mice per group. **(D)** Representative histopathological of the kidneys by PAS staining for *C. albicans*. Arrows indicate *C. albicans* filaments and yeast in the tissue. Model group: mice injected with *C. albicans* suspension and treated with normal saline daily. Control group: mice injected with normal saline, as the substitutes of *C. albicans* suspension and drug. AMP-17 (10 mg/kg) and FLC (10 mg/kg) group: mice injected with *C. albicans* suspension and daily treated with 10 mg/kg of AMP-17 and FLC, respectively. Analysis was performed by analysis of variance (ANOVA): Ns: *p* > 0.05, *p < 0.05, **p < 0.01, ***p < 0.001, compared with the model group. On the 8th days, six mice respectively, were sacrificed in each group for experiment.

To further confirm the protective capacity of AMP-17 *in vivo*, the survival rate of mice administered with AMP-17 in the murine model of disseminated candidiasis was examined. Survival was monitored for 15 days post-inoculation. Data from repeated experiments were combined. As expected, the untreated control began dying on day 3 post-inoculation, with 90% mortality (Figure 2B). While there was 75% survival in AMP-17-treated groups at the dose of 10 mg/kg, with no statistical difference when compared with the same dose of fluconazole having 80%.

In addition, disease severity was also examined according to the fungal burden in the kidneys dissected from the systemic candidiasis mice. As a result, intraperitoneal administration of AMP-17 at the dose of 10 mg/kg significantly reduced the fungal load in kidneys relative to the saline control (Figure 2C), as evidenced with improved survival with AMP-17 treatment. Concretely, 10 mg/kg of AMP-17 reduced *C. albicans* burden by 1.1 log10 (90%) fold in the murine kidneys post-7-day treatment. Likewise, the FLC administration (10 mg/kg) reduced the fungal burden by 1.2 log10 fold. Meanwhile, PAS staining of histopathological kidney sections was used to reflect the location of the colonized pathogen and tissue damaged by *C. albicans*. As shown in Figure 2D, large amount of the hyphae and pseudohyphae cells in the saline control group were colonized in the renal medulla, along with a large number of lymphocytes infiltrated at the follicular tissue. In contrast, there was an obvious *C. albicans* colonization found upon treatment with AMP-17. Nevertheless, a small number of fungal cells remained colonized in the renal tissue of fluconazole-treated mice. Above all, these results suggested that AMP-17 exhibits considerable potential as an antifungal agent *in vivo*.

### 3.3 AMP-17 attenuated the damage caused by *Candida albicans* and reduced inflammatory response

**Figure 3:**
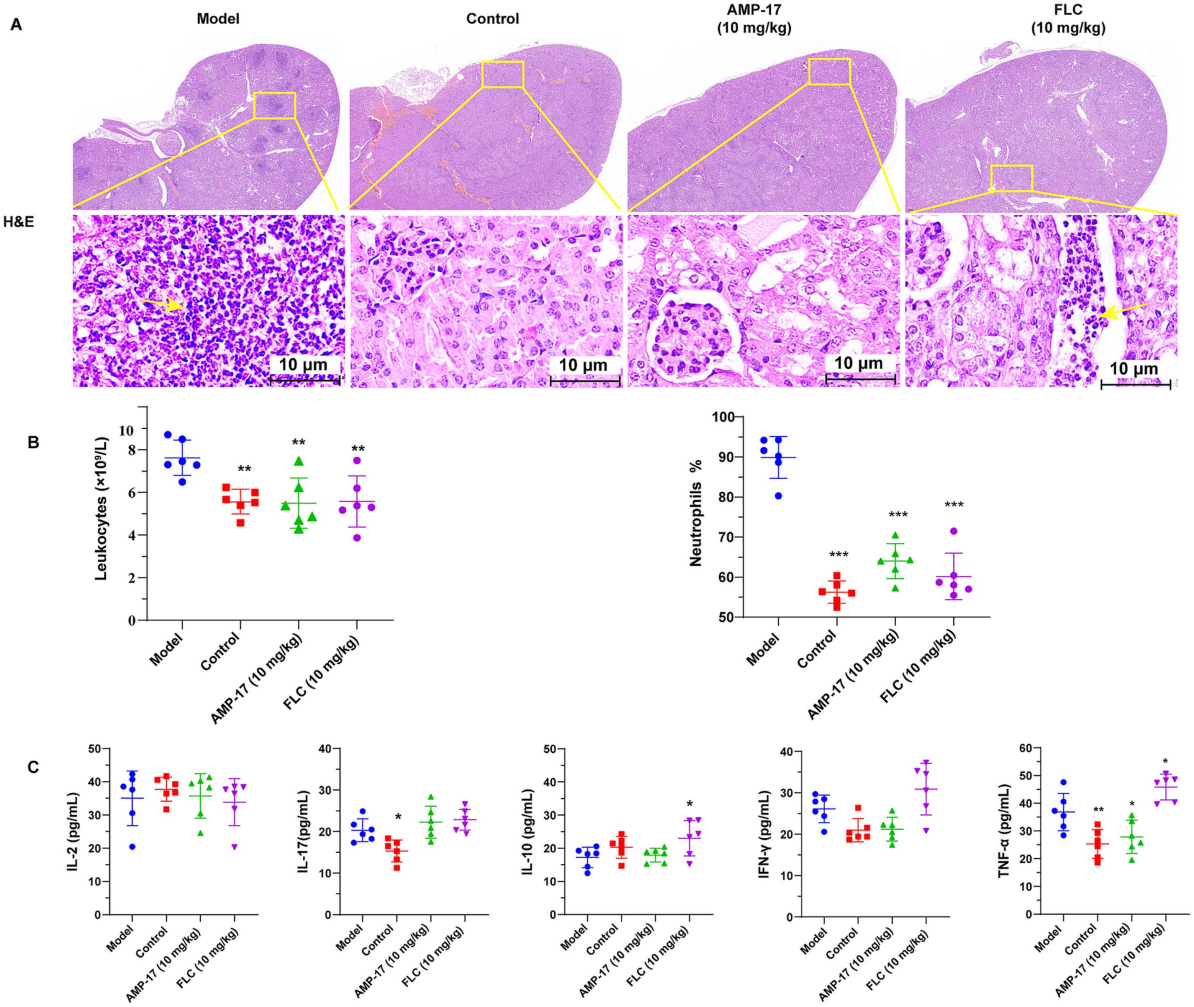
AMP-17 attenuated the damage of kidney and reduced inflammatory response. **(A)** Hematoxylin–Eosin (H&E) staining of histopathological kidney sections. The infiltration of leukocyte and lymphoid follicles are shown by the yellow arrow. **(B)** The leukocytes counts and the ration of neutrophils in plasma. **(C)** ELISA assay for the contents of cytokines containing IL-2, IL-17, IL-10, IFN-*γ*, TNF-α in mouse plasma. Analysis was performed by analysis of variance (ANOVA): Ns: *p* > 0.05, *p < 0.05, **p < 0.01, ***p < 0.001, compared with the model group. On the 8th days, six mice respectively, were sacrificed in each group for experiment.

Additionally, the histopathological features and inflammatory responses of kidneys exposed to *C. albicans* were evaluated using H&E staining tests. Diffuse small particle abscess was observed in both kidneys by eyes in the saline groups. Moreover, massive renal medullary necrosis and inflammatory infiltration occurred in the *C. albicans*-infected mice, as indicated by H&E staining. In addition, the renal cells exhibited extensive swelling with a significant increase in volume compared to the non-infected control group (Figure 3A). On the contrary, renal tissue necrosis and inflammatory responses were notably ameliorated, and the architecture of the kidney seemed to be intact after AMP-17 treatment, in line with the findings of survival and kidney fungal burden analyses.

Since AMP-17 was able to protect mice challenged by *C. albicans* from damage caused systemic candidiasis, we set out to test whether AMP-17 could lead to a slighter anti-*Candida* neutrophil response *in vivo* or a change of inflammatory factor concentrations in the mice subjected to *C. albicans.* Therefore, the count of peripheral neutrophils and the level of plasma cytokine and chemokine levels were measured. After the treatment of AMP-17 for 7 days, the level of leukocytes counts and the ratio of neutrophils in peripheral blood dramatically decreased (Figure 3B). Additionally, AMP-17 treatment resulted in a significant decrease in the concentration of proinflammatory cytokines (TNF-*α*), although the increase of anti-inflammatory cytokines (IL-10) or other proinflammatory cytokines (IFN-*γ*, IL-2, IL-17) were not statistically significant (Figure 3C). These results indicated the AMP-17 protected candidiasis mice from invasive damage by decreasing the levels of proinflammatory TNF-α, contributing to the reduction of leukocytes in peripheral blood. These results suggested AMP-17 has a potential therapeutic efficacy on systemic candidiasis, and can be considered as a promising alternative for clinical *C. albicans* infection in the future.

### 3.4 Synergistic effects of the combination of AMP-17 with fluconazole against *Candida albicans* biofilm formation

Although Fluconazole is the typical drug against *C. albicans* infections, long-term, high-dose use of fluconazole led to the emergence of drug-resistance strains, which poses a major challenge to the clinical practice of *C. albicans* infection (Zhang et al., 2020). AMP-17 exhibits significantly inhibitory effect on *C. albicans in vivo* (Yang L. et al., 2022; Ma et al., 2023) and *in vitro*, therefore it is valuable to further investigate whether the peptide has synergistic effects with fluconazole. To confirm the inhibitory efficacy of AMP-17 in combination with fluconazole *in vitro*, checkerboard microdilution methods were performed to examine for the antifungal and antibiofilm effects using the reference strain SC 5314 and 10 clinical isolates of *C. albicans*. For planktonic state, AMP-17 showed week-long combination activity with fluconazole against planktonic *C. albicans* (Supplementary Table S1). However, the combination of AMP-17 and fluconazole had synergetic or additive effects against *C. albicans* biofilms in most clinical isolates. As shown in Table 1, the FICI values of AMP-17 combined with FLC ranged from 0.28 to 0.53 *C albicans* reference strain and 9 other clinical isolates, suggesting a rather synergistic or additive effect. These data demonstrated that AMP-17, in most cases, potentiated the anti-biofilm susceptibility of FLC. Based on these data, a potential four-fold enhancement of the anti-biofilm effect of FLC in the presence of 32 μg/mL of AMP-17 could be expected. In summary, checkboard analysis ascertained the synergy effect between fluconazole and AMP-17 under the biofilm state of *C. albicans*.

**Table 1:** Synergistic effect of AMP-17 in combination FLC on *C. albicans* biofilm.

No	SMIC_80_			
	Agents alone		Combination	
	AMP-17 (μg/mL)	FLC (μg/mL)	AMP-17/FLC	FICI
SC 5314	128	>512	64/32	0.53 (PS)
16229	128	>512	64/32	0.53 (PS)
16138	128	>512	64/32	0.53 (PS)
16162	128	>512	64/32	0.53 (PS)
16228	128	>512	64/32	0.53 (PS)
16225	128	>512	128/32	1.03 (I)
16102	128	>512	32/32	**0.28 (S)**
16230	128	>512	32/32	**0.28 (S)**
16105	128	>512	64/32	0.53 (PS)
16214	128	>512	64/32	0.53 (PS)
16111	128	>512	64/32	0.53 (PS)

AMP-17, FLC, SMIC and FICI denoted antimicrobial peptide 17, fluconazole, the sessile minimum inhibitory concentration of the drug, and fractional inhibitory concentration index. SMIC was determined as 80% of inhibition of *C. albicans* biofilm formation compared to growth control. S, synergism (FICI < 0.5); PS, partial synergism (0.5 ≤ FICI < 1.0); AE, additive effect (FICI = 1.0); I, indifference (1.0 < IFCI ≤ 4.0).

**Figure 4:**
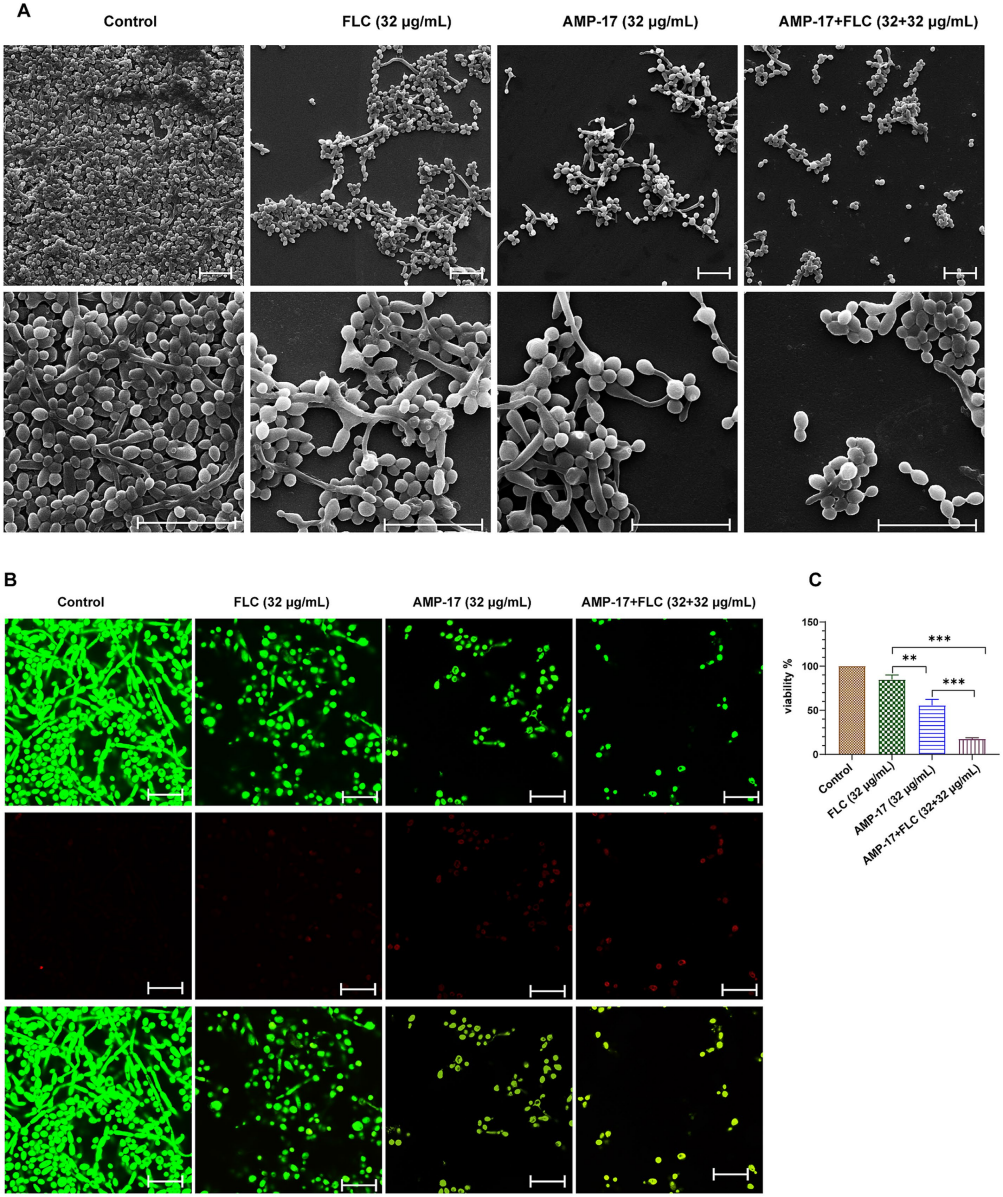
*In vitro* antibiofilm effect of AMP-17 in combination FLC on *C. albicans* (No. 16102) biofilm formation. **(A)** Scanning electron microscope (SEM) images of *C. albicans* biofilms formed on coverslips after different treatment for 12 h. Scare bar: 20 μm. **(B)** Confocal images of *C. albicans* biofilms obtained with live/dead staining (SYTO 9, green; PI, red) after different treatment for 12 h. Scare bar: 20 μm. **(C)** Cell viability of *C. albicans* biofilm detected by XTT assay. Ns: *p* > 0.05, *p < 0.05, **p < 0.01, ***p < 0.001, compared with the combined group.

Next, we evaluated *C. albicans* (clinical isolate 16230) biofilm morphology supplemented with AMP-17 or fluconazole for 12 h by scanning electron microscopy (SEM) (Figure 4A) and confocal laser scanning microscope (CLSM) (Figure 4B). As the results show, untreated *C. albicans* biofilm was composed of hyphal cells, and the biofilm was intact with a strong green fluorescence signal indicating living fungi. As expected, *C. albicans* in the presence of 32 μg/mL AMP-17plus 32 μg/mL FLC could not form biofilm and exhibited predominantly yeast form. However, the biofilms treated with single 32 μg/mL FLC consisted a mix of yeast form, pseudohyphal, and hyphal cells (Figures 4A,B). As well, the XTT assay showed that the inhibition of *C. albicans* biofilm viability was up to 83% after combined treatment (Figure 4C), but the treatment of single FLC at the concentration of 32 μg/mL reduced less than 30% biofilm viability, while AMP-17 at the same concentration decreased about 50% biofilm viability compared with the control. Thus, in light of the swift emergence of fluconazole-resistant strains within *C. albicans* isolates because of the prevalence of biofilm (Whaley et al., 2016), AMP-17 emerges as a prospective candidate to lower the MICs of conventionally applied antifungals and sustain their effectiveness in treating *C. albicans* biofilms.

### 3.5 Synergistic effects of AMP-17 in combination with fluconazole against preformed *Candida albicans* biofilm

**Figure 5:**
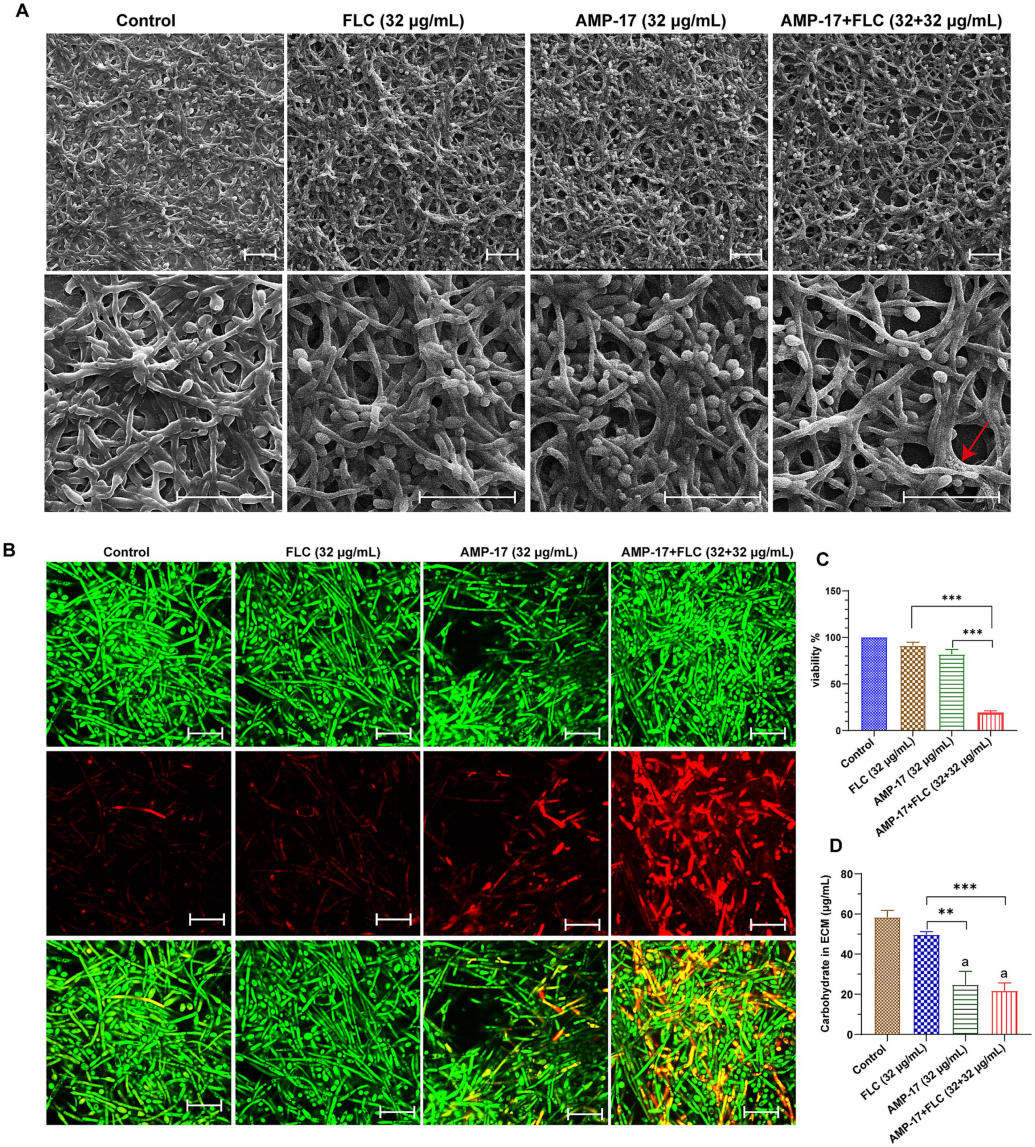
*In vitro* synergetic effect of AMP-17 in combination FLC on preformed *C. albicans* (No. 16105) biofilm. **(A)** Scanning electron microscope (SEM) images of mature *C. albicans* biofilms exposed to different drugs for 24 h. Scare bar: 20 μm. Red arrow indicated the surface of cell in biofilm was damaged and the cell content leaked outside. **(B)** Confocal images of preformed *C. albicans* biofilms on coverslips exposed to different drugs for 24 h. Images obtained with live/dead staining (SYTO 9, green; PI, red). Scare bar: 20 μm. **(C)** Cell viability of *C. albicans* biofilm detected by XTT assay. Ns: *p* > 0.05, *p < 0.05, **p < 0.01, ***p < 0.001, compared with the model group. **(D)** Qualification of carbohydrate in extracellular matrix in *C. albicans* biofilm exposed to different drugs for 24 h. Ns: *p* > 0.05, *p < 0.05, **p < 0.01, ***p < 0.001, compared with the combination group. “a” indicated *p* < 0.001 compared to with the control group.

Besides evaluating the combination effects of AMP-17 and FLC on *C. albicans* biofilm formation, we further investigated whether AMP-17 also had the synergetic effect on mature *C. albicans* biofilms when combined with FLC. The interaction of AMP-17 and FLC against mature *C. albicans* biofilm *in vitro* was tested, and the results was listed in Table 2. For two isolates, AMP-17 decreased the SMECs of FLC from >512 μg/mL to 32 μg/mL, with the FECI <0.5, indicating synergism between AMP-17 and FLC. As well, AMP-17 displayed an additive effect on two other isolates when combined with FLC, with their SMEC at least decreasing a gradient, respectively. For another four isolates, although the FECI were more than 0.5, the SMICs of FLC were also decreasing from >512 μg/mL to 32 μg/mL. Furthermore, XTT assays showed that the combination of 32 μg/mL AMP-17 and 32 μg/mL FLC inhibited more than 80% of preformed biofilms, while the equivalent FLC just reduced less than 10% biofilm viability, with at least a 16-fold increase in the susceptibility in eradication to mature *C. albicans* biofilm (Figure 5C). Also, morphological images demonstrated that AMP-17 plus FLC treatments loosen the biofilm structure, widen the spaces between the filamentous cells, and roughen biofilm surface, leading to lower biofilm stability and weaker resistance to environmental stress than single drug-treated groups (Figure 5A). Besides, more dead cells and lower cell viability were visualized in the preformed biofilms after AMP-17 plus FLC treatment, compared with single equivalent treatments or untreated ones (Figure 5B). The observation was congruent with the FECI values. Therefore, we confirmed that AMP-17 considerably improves the inhibitory efficacy of FLC on mature biofilm, with the best FECI value of 0.28, showing a synergistic effect on mature *C. albicans* biofilm.

**Table 2:** Synergistic effect of AMP-17 in combination FLC on preformed *C. albicans* biofilm.

No	SMEC_80_			
	Agents alone		Combination	
	AMP-17 (μg/mL)	FLC (μg/mL)	AMP-17/FLC	FECI
SC 5314	128	>512	32/32	0.28 **(S)**
16229	128	>512	128/>512	3.0 (I)
16138	128	>512	128/32	1.03 (I)
16162	128	>512	128/32	1.03 (I)
16228	128	>512	128/32	1.03 (I)
16225	128	>512	32/512	0.75 (PS)
16102	128	>512	64/512	0.75 (PS)
16230	>128	>512	>128/>512	2.0 (I)
16105	128	>512	32/32	0.28 **(S)**
16214	128	>512	128/32	1.03 (I)
16111	128	>512	128/>512	3.0 (I)

AMP-17, FLC, SMEC and FECI denoted antimicrobial peptide 17, fluconazole, the sessile minimum eradication concentration of the drug, and fractional eradication concentration index. SMEC was determined as 80% of eradication preformed *C. albicans* biofilm compared to growth control. S, synergism (FECI < 0.5); PS, partial synergism (0.5 ≤ FICI < 1.0); AE, additive effect (FICI = 1.0); I, indifference (1.0 < IFCI ≤ 4.0).

For exploring the action of the synergistic effect of AMP-17 plus FLC, carbohydrate in extracellular matrix (ECM) of *C. albicans* biofilms was quantified. The ECM, essential for shielding enclosed cells from approaching antifungal agents, serves as the backbone of every biofilm community (Karygianni et al., 2020). And carbohydrate, as the main composition of ECM, takes a ratio of approximately 15% in ECM (Gulati and Nobile, 2016). As depicted in Figure 5D, a considerable decrease in carbohydrate of *C. albicans* biofilm ECM was observed after AMP-17, ranging from 58.33 μg/mL to 24.67 μg/mL. Importantly, the carbohydrate in the AMP-17 plus FLC-treated group decreased approximately 63%, with a similar efficacy of AMP-17. In contrast, the equivalent FLC treatment only reduced less than 15% carbohydrate, having indifference with the control. Thus, the inhibitory effect of the combined treatment on carbohydrate in biofilm ECM mainly depended on the action of AMP-17. In a word, AMP-17 had a synergistic effect on eradicating mature *C. albicans* biofilm when combined with FLC, mostly due to the inhibitory efficacy of the peptide on carbohydrate production in biofilm ECM.

### 3.6 Antifungal effect of AMP-17 in combination with fluconazole against *Candida albicans in vivo*

**Figure 6:**
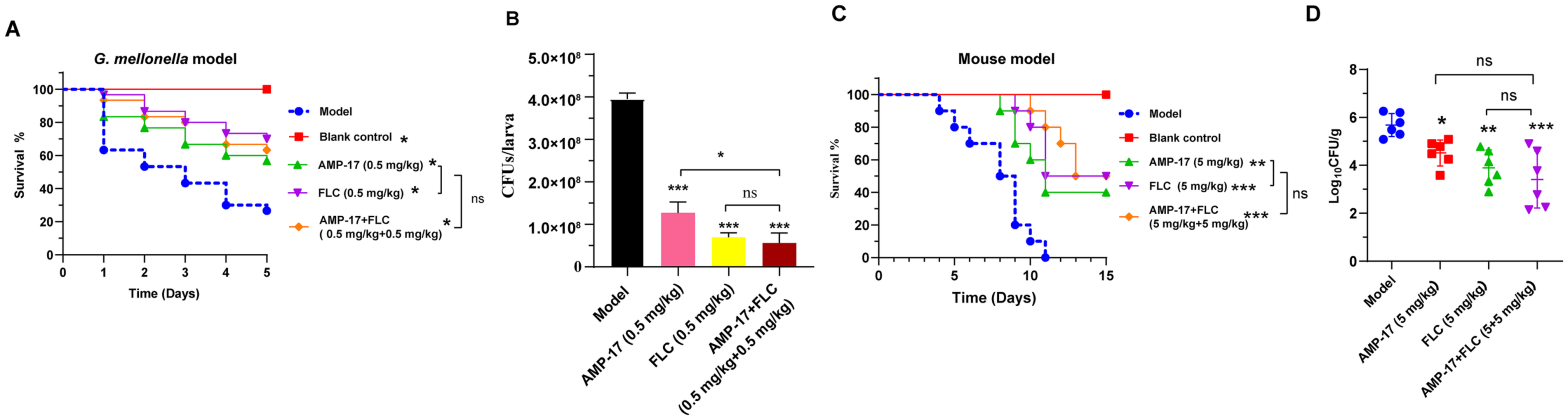
The combination effect of AMP-17 plus FLC in *C. albicans* infected *G. mellonella*. The survival **(A)** and fungal loads **(B)** in the *G. mellonella* larvae challenged with 0.5 × 10^6^ *C. albicans/*larva and the therapeutic effect of different treatment including AMP-17 (0.5 mg/kg), FLC (0.5 mg/kg) and AMP-17 plus FLC (0.5 mg/kg + 0.5 mg/kg). The survival **(C)** and fungal loads of kidney **(D)** in mice challenged with 2 × 10^6^ *C. albicans*/mouse by intravenous injection and the therapeutic effect of different treatment including AMP-17 (5 mg/kg), FLC (5 mg/kg) and AMP-17 plus FLC (5 mg/kg + 0.5 mg/kg). The Kaplan–Meier survival curves of *G. mellonella* monitored for 5 days post-inoculation and mice for 15 days. *N* = 30 *G. mellonella* larvae or 10 mice per group. Survival rate of mice was carried out by the log-rank test. The analysis of *C. albicans* colony counts were performed by analysis of variance (ANOVA): Ns: *p* > 0.05, *p < 0.05, **p < 0.01, ***p < 0.001, compared with the model group.

In view of the excellent synergistic effect of AMP-17 in combination with FLC *in vitro*, we further attempted to explore the combination effect of AMP-17 plus FLC against invasive Candidiasis *in vivo*. From the *C. albicans* infected-*G. mellonella* model, the survival curve displayed that the combination of AMP-17 and FLC (0.5 mg/kg + 0.5 mg/kg) did not significantly enhance the survival of infected *C. mellonella* larvae compared to equivalent AMP-17 or FLC treatments but did protect 63% of larvae from mortality within 5 days post-treatment (Figure 6A). With regards to the fungal burden of the infected *G. mellonella*, all treatments could dramatically reduce *C. albicans* colony counts over 65%, and the decrease of CFUs in each larva exposed to AMP-17, FLC, and AMP-17 plus FLC was 2.69 × 10^8^, 3.25 × 10^8^, and 3.39 × 10^8^, respectively (Figure 6B). From the mice subjected to invasive candidiasis, single or combined treatment could infectively improve the survival and reduce fungal burden in *C. albicans*-infected mice. The treatment of AMP-17 plus FLC (5 mg/kg + 5 mg/kg) increased 50% survival of disseminated candidiasis mice, with no significant difference with AMP-17 of 40% survival and FLC of 50%. However, the survival curve showed the median survival of AMP-17 + FLC, AMP-17, and FLC was 14, 11, and 13 days, compared to the model group 8.5 days (Figure 6C). In addition, the treatment of AMP-17 + FLC, FLC, and AMP-17 all significantly reduced the *C. albicans* colony counts in kidneys up to more than 90%, ranging from 4.82 × 10^5^ to 2.58 × 10^3^, 7.85 × 10^3^, and 3.26 × 10^4^ CFU/g, respectively (Figure 6D). Thus, *in vivo* experiments demonstrated AMP-17 in combination with fluconazole showed similar antifungal effects against disseminated candidiasis in *C. albicans* with the treatment of AMP-17 alone.

## 4 Discussion

*Candida albicans* is a primary fungus responsible for mucous infection and invasive candidiasis (Pappas et al., 2018), resulting in great physical and mental pain as well as economic loss. The treatment of fungal infections is hindered by extended treatment protocols and the prevalence of antifungal drug resistance in numerous fungal genera (Pappas et al., 2018), and the low number of available antifungal agents worsens the serious situation (Voltan et al., 2016). Hence, there is an urgent need for innovative strategies to address the resistance issue. Recently, AMPs have emerged as promising antimicrobial agents, demonstrating broad-spectrum activity and a reduced likelihood of pathogen resistance (Wang et al., 2012). Our previous study has demonstrated that AMP-17 possessed antifungal activity against *C. albicans* either in planktonic form or in the highly virulent forms such as hypha and biofilm (Sun et al., 2022; Yang L. et al., 2022; Ma et al., 2023). These *in vitro* significant findings paved a promising way for exploring whether AMP-17 had effective ability against *C. albicans* infections *in vivo*. Herein, AMP-17 was proved to have therapeutic effect against systemic candidiasis in the wax moth and murine model of disseminated candidiasis induced by *C. albicans*. Besides, the peptide presented significant synergistic efficacy with fluconazole against biofilms of *C. albicans.*

*Candida* spp. can cause persistent inflammatory processes like vaginitis, stomatitis, and paronychia and is particularly prevalent in cancer patients undergoing cytostatic drug treatment, immunocompromised individuals, or those with severe burns. The outcomes of unrestrained infection and inflammation can be so serious that they cause organ failure and ultimately death (Gentile et al., 2012). Consequently, an effective treatment should not only eradicate *Candida* spp. but also dampen the excessive inflammation (Garcia-Rubio et al., 2019). Similar to humans, mice infected with *C. albicans* also undergo severe pain as a result of candidiasis. The weight and survival rate of mice subjected to disseminated candidiasis dramatically decline, and an intense inflammatory response has occurred in the mice model and *G. mellonella* model of systemic candidiasis. In contrast, AMP-17 treatment showed an obvious survival benefit and decrease in fungal colonization (Figures 1, 2). Meanwhile, H&E and PAS staining assays demonstrated that kidney injury and inflammation reduced significantly with the treatment of AMP-17. These results confirmed that AMP-17 demonstrated protective effects in both the wax moth and murine model of disseminated candidiasis, enhancing the clearance of *C. albicans* and consequently reducing kidney damage.

Although the inflammatory response is crucial for clearing infections and promoting healing, uncontrolled overactivation can be detrimental, further complicating the healing process (Guo and Dipietro, 2010; Pathakumari et al., 2020). Reportedly, besides direct antimicrobial properties, AMPs can enhance immunity and bridge adaptive and innate immunity by acting as immunomodulators on host cells (Nagaoka et al., 2020). Macrophages and neutrophils are key effector cells in the innate immune system against *C. albicans* (Kumar and Sharma, 2010; Pappas et al., 2018). Moreover, the ability of *Candida albicans* to transition between filamentous hyphae and single-celled yeast is intrinsically tied to the pathogenicity of the organism. The solitary form of yeast is small and easily taken up by neutrophil phagocytosis. On the other hand, the hyphal, pseudohyphal, and filamentous forms are larger than neutrophils and cannot be cleared by swallowing. As a result, in order to neutralize the hyphae, neutrophils must employ alternate effector mechanisms. Our data suggested that AMP-17 treatment inhibited an excessive inflammatory response, as evidenced by the disappearance of leukocyte infiltration and the formation of lymphoid follicle-like structures induced by *C. albicans* in the kidney tissue, relative to untreated mice (Figure 3A). However, the level of the leukocyte counts and neutrophil ratio in plasma in AMP-17-treated groups was already higher than the control group (Figure 3B). That is not surprising, since it was previously reported that neutrophils, a key number of host innate immunity, take an important part in clearing the invading *C. albicans* in the way of neutrophil extracellular traps (NETs) that subsequently kill fungi (He et al., 2022). Therefore, AMP-17 likely plays a positive role in aiding host cells to kill fungi without causing inflammatory injury, helping to maintain the balance of innate immunity cells. On the other hand, our results indicated that AMP-17 elevated cytokine levels, including gamma interferon (IFN-*γ*) and interleukin-2 (IL-2) that stimulate Th1 cells and CD8 T cells, along with interleukin-17 (IL-17) implicated in *C. albicans* infections (Li et al., 2017). But in contrast to untreated mice, the level of tumor necrosis factor alpha (TNF-*α*) in plasma of the infected mice significantly decreased after AMP-17 treatment. This suggests that AMP-17 induces an anti-inflammatory response by mainly downregulating pro-inflammatory cytokines TNF-α, besides IFN-γ, IL-17, and IL-2. Thus, AMP-17 exerted its therapeutic effect against systemic candidiasis in a murine model, probably through inhibition of fungi colonization as well as an anti-inflammatory response.

Due to the biofilm acting as a barrier to antifungal drugs and phagocytes, some first-line clinical antifungal drugs, like azoles and echinocandins, exhibit reduced sensitivity in treating associated invasion. Antifungal agents capable of reducing biofilm microbes show promise in decreasing mucus colonization and blood dissemination (Ma L. et al., 2020; Miao et al., 2022). Previous studies showed that AMP-17 exerted effectively anti-biofilm effects and hyphal growth inhibition (Ma H. et al., 2020; Sun et al., 2022). Given that combining drugs can yield positive clinical outcomes and enhance individual agent efficacy, the combination of AMP-17 and fluconazole was likely to heighten each other’s sensitivity against *C. albicans* biofilms. Here, we confirmed that AMP-17 synergistically enhanced the efficacy of FLC against *C. albicans* in both biofilm formation and mature biofilms. When FLC combined with AMP-17 against *C. albicans* biofilms, its SMIC_80_ and SMEC_80_ ranges declined from > 512 μg/mL to 32 μg/mL (Tables 1, 2), achieving a total synergy rate of up to 80% for biofilm formation (Table 1) and close to 40% for preformed biofilm eradication (Table 2). Moreover, the combination of AMP-17 and FLC hardly showed synergistic efficacy in the planktonic state, possibly due to the high sensitivity of the selected strains to fluconazole (An et al., 2023; Supplementary Table S1). Above all, AMP-17 did not only show superior efficacy against *C. albicans* biofilm than fluconazole but also increased the sensitivity of fluconazole against biofilm at least fourfold.

For the synergistic mechanism, morphogenesis of *C. albicans* was necessary to be tested. As *C. albicans* has morpho-plasticity as a yeast, pseudo-hyphae, and hyphae form, hyphal morphology is crucial in the biofilm formation process, creating too dense network structures to permeate antifungal agents. Thus, abnormal yeast-to-hyphae transformation can suppress *C. albicans* biofilm associated infections. In the current study, FLC yields a week anti-biofilm effect on all *C. albicans* strains (SMIC_80_ and SMEC_80_>512 μg/mL) *in vitro*, and cannot block hyphal development effectively. But the combination of AMP-17 and fluconazole kept most fungi in the yeast form and further progressed to hyphal development (Figures 4A,B). Although the exact action of hyphal inhibition by AMP-17 combined with FLC remained unknown yet, transcriptional profile analysis suggested that AMP-17 can interfere in MAPK-mediated signal transduction reflected by downregulation effectors such as ALS3 (adhesion), TEC (hyphal inducer), ECE1 (yeast elongation), and RFG1 (yeast to hyphal transformation) (Sun et al., 2022).

For preformed biofilms, AMP-17 in combination with FLC reduced the stability of preformed biofilms, with the features of a higher rate of cell lysis, a much looser constitution, and leakage of extracellular matrix (ECM). Considering ECM is essential for yeast cells to develop a multicellular, structured community, it is crucially necessary for an antifungal to disrupt ECM in order to ensure it enter. Biochemical analysis clarified that the carbohydrate was decreased in the preformed biofilm in the presence of AMP-17 and AMP-17 plus FLC, while no change was found in FLC alone treated *C. albicans* mature biofilms (Figure 5D). Carbohydrate, the basis of exopolysaccharide secreted by biofilms, plays an essential role in biofilm establishment, colonization, and penetration of hyphal cells deep into tissue (Lee et al., 2016). Saima Muzammil found that the content of protein, extracellular polysaccharide, and eDNA in the extracellular matrix of *Acinetobacter baumannii* was reduced after aluminum oxide nanoparticle treatment (Muzammil et al., 2020). Another study found the extracellular polysaccharide of *F. solani* biofilm content was decreased by 83.36% when *F. solani* was cultured with 1,8-cineole for 72 h (Zhang et al., 2022). We hypothesized the antibiofilm ability of AMP-17 plus FLC was associated with affecting the composition of ECM in a similar way with 1,8-cineole. Interestingly, the results showed that AMP-17 in combination with FLC could inhibit extracellular carbohydrates. However, any change in its composition can reflect upon the viability of the cells present in a given biofilm architecture, and quantitative analysis of ECM’s components is the crucial parameter that provides important biochemical evidence regarding the antibiofilm activity of the peptide. Therefore, it would be highly beneficial to investigate the other components of the extracellular matrix and explore the regulatory pathway in subsequent studies.

Moreover, the *in vivo* interaction of AMP-17 in combination with FLC was also estimated in *G. mellonella* and mice models of systemic candidiasis induced by *C. albicans*. Survival curve and fungal burden analysis demonstrated AMP-17 in combination with FLC can significantly improve the mortality rate of *C. albicans*-infected larvae or mice and reduce the pathogen colonization of infectious ones, but the combined treatment did not show an obvious difference in therapeutic efficacy compared to the treatment of AMP-17 alone. Interestingly, fluconazole presents a low sensitivity against *C. albicans* biofilm when experimented *in vitro*, whereas it *in vivo* showed a significant reduction in CFUs in the kidneys. The variation may stem from susceptibility tests assessing antifungal activity under static conditions, which do not accurately replicate *in vivo* situations where numerous factors, such as the underlying condition and immunological status of the animal model, may impact intrinsic antifungal properties (Rex et al., 1995). Additionally, *in vivo* experiments, the unideal synergism of AMP-17 in combination with FLC might be ascribed to the inadaptable concentration ratio of the two drugs or suggest there was no synergistic effect between AMP-17 and fluconazole on planktonic *C. albicans* infection *in vivo*. In the future, we need to construct a suitable biofilm model of *C. albicans* infection in order to confirm the synergism of AMP-17 in combination with FLC against *C. albicans* biofilm *in vivo.*

## 5 Conclusion

In summary, we evaluated the antifungal activity of AMP-17 against *C. albicans in vivo* and the synergistic anti-biofilm of the peptide plus FLC. Our study demonstrated that AMP-17 protected *G. mellonella* and mice from systemic candidiasis and decreased fungal burden *in vivo*. Further investigation into the mode of action confirmed that AMP-17 improved *C. albicans* infected-mice recovery by regulating the balance of the inflammatory response. Moreover, the present study demonstrated that AMP-17 exhibited a synergistic effect against biofilm formation and preformed biofilm when combined with FLC *in vitro*. Although further studies are needed to clarify the underlying mechanism of these effects, the *in vivo* effect of AMP-17 on systemic candidiasis in both *G. mellonella* and mouse model, together with the synergistic anti-biofilm effect in combination with FLC, highlights that AMP-17 has a promising practical value for the treatment of infections caused by *C. albicans*.

## Data Availability

The original contributions presented in the study are included in the article/Supplementary material, further inquiries can be directed to the corresponding author.
