# Supplementary material for: Antimicrobial peptide AMP-17 induces protection against systemic candidiasis and interacts synergistically with fluconazole against Candida albicans biofilm
Source: Front Microbiol. 2024 Nov 1;15:1480808. doi: 10.3389/fmicb.2024.1480808 (PMC11564183; doi:10.3389/fmicb.2024.1480808)
Supplement: Supplementary file 1 [file Table_1.docx]

Supplementary Material

**Supplementary table 1 Combination activity of AMP-17 and fluconazole on planktonic *C. albicans***

|  | **MICs** | | | |
| --- | --- | --- | --- | --- |
|  | **Agents alone** | | **Combination** |  |
| **No.** | **AMP-17 (μg/mL)** | **FLC (μg/mL)** | **AMP-17/FLC** | **FICI** |
| SC 5314 | 16 | 2 | 16/1 | 1.5 (I) |
| 16229 | 16 | 2 | 16/1 | 1.5 (I) |
| 16138 | 16 | 2 | 16/1 | 1.0 (AE) |
| 16162 | 16 | 4 | 8/2 | 1.5 (I) |
| 16228 | 16 | 2 | 8/2 | 2.0 (I) |
| 16225 | 16 | 2 | 16/2 | 1.5 (I) |
| 16102 | 16 | 2 | 16/1 | 1.5 (I) |
| 16230 | 16 | 2 | 16/1 | 1.5 (I) |
| 16105 | 16 | 4 | 16/2 | 1.5 (I) |
| 16214 | 16 | 2 | 16/2 | 1.5 (I) |
| 16111 | 16 | 2 | 16/2 | 2.0 (I) |

AMP-17, FLC, MIC and FICI denoted antimicrobial peptide 17, fluconazole, the MIC of the drug. And fractional inhibitory concentration index. MIC was determined as 80% of inhibition of fungal growth compared to growth control. S, synergism (FICI<0.5); AE, additive effect (FICI=1.0); I, indifference (1.0<IFCI≤4.0).
